# Double-Crosslinked Soy Protein Isolate Film with Improved Antibacterial and Antioxidant Performances via Introducing Phytic Acid for Packaging Beef Tallow and Chicken

**DOI:** 10.3390/polym18172131

**Published:** 2026-09-01

**Authors:** Hairu Wang, Zihao Ren, Xiling Cheng, Ziyan Peng, Lijuan Wang

**Affiliations:** Key Laboratory of Bio-Based Material Science and Technology of Ministry of Education, Northeast Forestry University, Hexing Road 26, Harbin 150040, China; 18766259553@163.com (H.W.); lsm3144522571@163.com (Z.R.); 17603448533@163.com (X.C.); xxaoo@nefu.edu.cn (Z.P.)

**Keywords:** SPI film, phytic acid (PA), antibacterial, antioxidant, heat sealing

## Abstract

In order to improve antibacterial and antioxidant performances, phytic acid (PA) was incorporated into soy protein isolate (SPI) film crosslinked with tara tannins (TTs) and dialdehyde cellulose nanofibers (DACNFs). The resulting PDTSPI (PA/DACNF/TT/SPI) film exhibited enhanced physical and UV-Vis shielding and antioxidant properties. At 15% PA content, the tensile strength (TS) reached 5.17 MPa and the elongation at break (EB) increased to 101.15%, showing the comprehensive mechanical performance. After the addition of PA, the composite films displayed negligible light transmittance at wavelengths under 460 nm, with visible light transmittance reduced to less than 40%. And the DPPH radical scavenging rate increased to 49.2%, demonstrating strengthened antioxidant activity of PDTSPI films. Notably, increasing PA content led to improved antibacterial effects against *Escherichia coli* (*E. coli*) and *Staphylococcus aureus* (*S. aureus*), with the inhibition rate of PDTSPI-12 and PDTSPI-15 films for *S. aureus* achieving 100%. The peroxide value (POV) of beef tallow and total viable count (TVC) of chicken packaged with PDTSPI-15 were only 0.007 g/100 g and 2.17Log CFU/g, respectively—both significantly lower than those packaged with pure SPI film, which demonstrates the potential of PDTSPI film for packaging oily foods and meat products.

## 1. Introduction

Nowadays, plastic packaging has become indispensable in our daily life [1]. However, its non-degradable characteristic leads to serious and irreversible environmental problems [2]. Moreover, plastic fragments enter oceans and marine organisms, eventually posing risks to human health through the food chain [3]. To address this issue, biomass-based films with versatile properties [4], have attracted widespread attentions as ideal alternatives to plastic films for food preservation [5].

Among various biopolymer-based materials, SPI film possesses great gas barrier properties, UV-blocking capacity and excellent heat-sealing performance [6,7]. Despite its potential in food packaging field, SPI film suffers from limited antioxidant and antibacterial activities [8]. The introduction of Xanthoceras sorbifolia husk caused improvement in the antioxidant capacity of SPI films [9], and a strategy was illustrated that tea polyphenols and carvacrol were used to develop antimicrobial SPI films [10]. In our previous study, TT and DACNF were used to crosslink SPI to enhance antioxidant [11] and antibacterial activities of the film [12]. However, these two main properties remained unsatisfactory and required further improvement.

**Figure 1:**
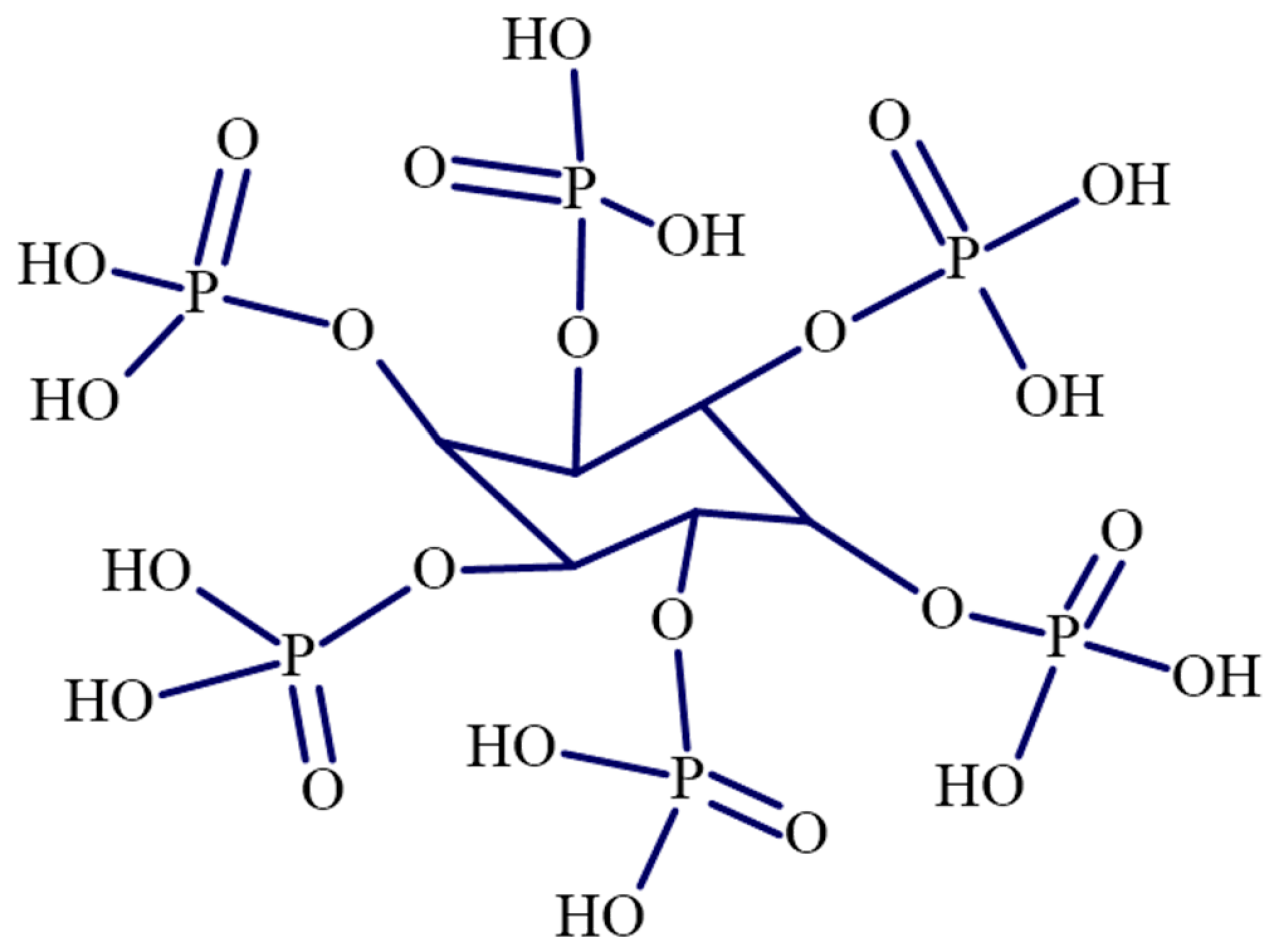
Chemical structure of PA.

In recent years, numerous antioxidant and antibacterial agents have been applied in food packaging to prolong shelf time [13]. But many synthetic agents leave toxic residues, harming both the environment and human health. Therefore, natural agents have been extensively investigated due to their abundance, low cost and eco-friendliness [14]. Phytic acid (PA), a natural, non-toxic and renewable substance with antioxidant and antibacterial activity as well as biocompatibility [15], can serve as an ideal candidate to improve antioxidant and antibacterial activities of the films. The detailed chemical structure of PA has been depicted in Figure 1. Rice bran purified phytic acid was a suitable and efficient natural antioxidant in mechanically deboned chicken meat [16]. And a chitosan–phytic acid complex was synthesized to produce a novel green corrosion inhibitor with impressive antimicrobial and anti-biocorrosion properties [17]; as well, research has been reported that phytic acid was combined with κ-carrageenan and carboxylated cellulose nanofibril to fabricate antibacterial films [18]. In this work, PA was introduced into the DACNF/TT/SPI system, endowing composite film with superior antibacterial capacity, improved antioxidant, UV-Vis blocking and mechanical properties.

Heat sealing is an essential technology in food packaging owing to its simplicity and high cost-effectiveness [19]. Compared with other polymers, the heat transition temperature of SPI film is much lower than its decompose temperature [20], making heat sealing highly suitable for fabricating packaging pouches. For instance, blueberries were heat-sealed in diatomite/thymol/SPI films, remaining fresh for five days [21]. To our knowledge, no research has yet reported the heat-sealing conditions for PDTSPI films.

In this study, novel composite films were fabricated by integrating PA into a matrix composed of DACNF, TT, and SPI. The resultant PDTSPI films exhibited improved antibacterial and antioxidant capabilities and enhanced UV-Vis light shielding, while simultaneously achieving a more balanced mechanical strength. The optimal heat-sealing temperature was determined through differential scanning calorimetry (DSC) and scanning electron microscopy (SEM). To evaluate the practical application, the films were employed to package beef tallow and chicken, and their packaging performance was assessed by monitoring peroxide value (POV) and total viable count (TVC) throughout the storage. This work provides a new idea for developing antioxidant and antibacterial packaging films derived entirely from renewable resources.

## 2. Materials and Methods

### 2.1. Raw Materials

SPI (more than 90% protein) was purchased from Harbin High Technology Soy Protein Co., Ltd. (Harbin, China). Tara tannins were acquired from Sichuan Tingjiang Deyang Technology Co., Ltd. (Deyang, China). DACNF was prepared by oxidizing cellulose nanofibers (CNF, Wuhan Huaxiang Kejie Biotechnology Co., Ltd., Wuhan, China) with sodium periodate (NaIO_4_, from Tianjin Kemiou Chemical Reagent Co., Ltd., Tianjin, China). Analytical grade glycerol was provided by Tianjin Yongda Chemical Reagent Co., Ltd. (Tianjin, China). PA was supplied by Tianjin Wengjiang Chemical Reagent Co., Ltd. (Tianjin, China). 1,1-diphenyl-2-picryl-hydrazyl (DPPH) was procured from Sigma-Aldrich Co., Ltd. (Darmstadt, Germany). *Escherichia coli* (*E. coli*) and *Staphylococcus aureus* (*S. aureus*) were obtained from Qingdao Hope Bio-Technology Co., Ltd. (Qingdao, China). Beef tallow and chicken were sourced from the Biyoute market in Harbin, China.

### 2.2. Preparation of PA/DACNF/TT/SPI Films

First, 5.90 g of SPI was dissolved in 100 mL distilled water, followed by the addition of 2.95 g glycerol, and the mixture was stirred at 85 °C for 10 min. DACNF was fabricated via oxidizing the -OH on C2 and C3 of glucose in nanocellulose fibers to aldehyde groups [22]. Subsequently, TT (8% *w*/*w* relative to SPI mass) and DACNF (10% *w*/*w* relative to SPI mass) were introduced, after which the solution was sonicated for 3 min and stirred for another 5 min. Then, PA (3%, 6%, 9%, 12% and 15% of SPI mass) was added into the mixture. and the pH was adjusted to 10 under continuous stirring until homogeneous. Ultimately, the solution was poured into a mold and dried at 55 °C for 12 h. The obtained films were labeled as PDTSPI-3, PDTSPI-6, PDTSPI-9, PDTSPI-12 and PDTSPI-15. Meanwhile, for comparison, control groups were also prepared as follows: SPI film (pure SPI), TSPI film (8% TT of SPI mass) and DTSPI film (8% TT and 10% DACNF of SPI mass).

### 2.3. Characterization of Films

#### 2.3.1. Morphology and Structure of the Films

The films were cryofractured by immersing in liquid nitrogen and pasted onto conductive adhesive tapes, coated with an ultra-thin layer of gold; then, the samples were observed on the surface and the cross-section using a QUANTA 200 scanning electron microscope (Philips-FEI Co., Ltd., Hillsboro, OR, USA) at an accelerating voltage of 5 kV.

For FTIR analysis, the films (2.0 cm × 2.0 cm) were scanned using a Nicolet iS50 FTIR spectrometer (Thermo Fisher Scientific Co., Ltd., Waltham, MA, USA) over the range of 4000 to 500 cm^−1^ with a resolution of 4 cm^−1^.

#### 2.3.2. Optical Performance

The optical transmittance of the films (40.0 mm × 20.0 mm) was measured by UV–Vis spectroscopy (Shimadzu, Kyoto, Japan) over wavelengths of 200–800 nm.

#### 2.3.3. Mechanical Properties

Films (15 mm × 80 mm) were conditioned at 53% relative humidity (RH) for 12 h. Five random positions on the films were selected for thickness measurement with a D-C11ZXBS instrument (Mitutoyo, Kawasaki, Japan), and the average value was subsequently calculated.

According to standard GB/T 1040.3-2006 [23], the tensile strength (TS) and elongation at break (EB) were determined using an XLW-PC tensile tester (Jinan, China) with a 50.0 mm initial grip separation and 300 mm/min cross-head speed. Values were averaged over five measurements. The TS and EB were calculated according to the following Formulas (1) and (2):

(1) $$\;\;\;\;\;TS\left(MPa\right)=\frac{F}{W\times THK}$$

where *F* (N) is the breaking force (N), *W* (mm) is the width, and *THK* (mm) is the thickness of the film.

(2) $$\;\;\;\;\;EB\left(\%\right)=100\times \frac{L-L_{0}}{L_{0}}$$

where *L*_0_ (mm) is the initial length, and *L*_0_ (mm) is the length at break.

#### 2.3.4. Water Vapor and Oxygen Permeability

According to ASTM E96 [24], the modified gravimetric method was taken to measure water vapor permeability (WVP, g·m^−1^·s^−1^·Pa^−1^). Films were cut into circles with diameters of 6.0 cm, then twenty locations on the samples were chosen randomly to test the thickness. The films were sealed on the top of weighing bottles preloaded with 23.0 g dried anhydrous CaCl_2_. After balancing the humidity at 0% RH for 12 h, the initial mass of each bottle was weighed, and then the bottles were transferred to a constant moisture chamber at 75% RH (saturated NaCl solution) and 25 °C. Mass variation was recorded at intervals and the WVP was calculated by Formula (3):

(3) $$\;\;WVP\left(g\cdot m^{-1}\cdot s^{-1}\cdot {Pa}^{-1}\right)=\frac{∆M\times T}{∆t\times A\times P}$$

where Δ*M* (g) is the change of weight at intervals; *T* (mm) is the film thickness; Δ*t* (s) is the time interval; *A* (m^2^) is the water vapor passing area; and *P* (Pa) is the osmotic pressure of a saturated NaCl solution.

According to standard GB/T 19789-2021 [25], oxygen permeability (OP, cm^3^·mm^−2^·day^−1^·Pa^−1^) of the films was determined by an OX/230 oxygen permeability tester (Labthink Instruments Co., Ltd., Jinan, China). The films (9.0 cm × 10.0 cm) were placed in the measuring chamber, and the ambient testing conditions were set as follows: temperature of 25 °C, relative humidity (RH) of 0%, test area of 50 cm^2^, nitrogen flow rate of 10 ± 1 mL/min, and oxygen flow rate of 60 ± 10 mL/min.

#### 2.3.5. Antioxidant Activity

Then, 0.1 g of the films was immersed in 10 mL of 10% ethanol solution, shaking at 25 °C for 12 h. Then, 1 mL of the release solution was mixed with 4 mL of DPPH radical solution (25 mg/L); then, the mixture was reacted for 30 min in the dark, and its absorbance was tested at 517 nm. In the control experiment, 95% ethanol replaced the release solution, and the above procedures were repeated accordingly. The DPPH scavenging activity (SA) was calculated by Formula (4):

(4) $$SA\left(\%\right)=\frac{A_{c}-A_{s}}{A_{c}}\times 100$$

where *Ac* and *As* are the absorbance of the control and testing sample solution, respectively.

#### 2.3.6. Antibacterial Activity

The antibacterial activity was evaluated by the plate counting method [15]. *E. coli* and *S. aureus* were chosen as the experimental bacterial strains; as well, the optimal concentration of bacterial suspension was 10^4^ CFU/mL. After mixing 1 mL of bacterial suspension with 9 mL of PBS, 1.0 g of the sample was immersed in the resulting solution and agitated at 37 °C for 3 h. Subsequently, 200 μL of the mixture was spread on nutrient agar medium uniformly and incubated at 37 °C for 24 h. The number of colonies formed on the agar was recorded to assess the antibacterial activity of the films.

### 2.4. Heat Sealing of the Films

Two film strips (100 mm × 15 mm) were heat-sealed using an AT-RF Heat Seal Tester (Anmc, Jinan, China). After conditioning at 53% RH for 12 h to achieve moisture balance, the heat-sealing strength was measured using a tensile tester with a 50 mm grip separation and 2 mm/s upward moving speed. Each sample was measured repeatedly at least five times.

The differential scanning calorimetry (DSC) curves were examined using a Q2000 differential scanning calorimeter (TA Instruments, New Castle, DE, USA). Samples were loaded into aluminum pans, then sealed and scanned at a heating rate of 10 °C/min. The sealing effectiveness was verified by SEM images.

### 2.5. Packaging Beef Tallow and Chicken with the Films

The film rectangles (10.5 cm × 7.5 cm) were heat-sealed into pouches containing 5 g of beef tallow. Control groups included unpackaged beef tallow and polyethylene (PE)-packaged samples. The effect of the film was evaluated by testing the peroxide value (POV) of beef tallow during storage in accordance with GB 5009.227-2023 [26].

Samples were sterilized under ultraviolet light for 45 min. Chicken (already inoculated with the experimental strain) was sealed in pouches from the films and its total viable count (TVC) was measured every 3 days according to GB 4789.2-2022 [27]. The colony-forming units per gram (Log CFU/g) were recorded as the result.

### 2.6. Data Processing and Statistical Analysis

Experimental data were analyzed using SPSS 20.0 (SPSS Inc., Chicago, IL, USA) and expressed as mean ± standard deviation. One-way ANOVA combined with Tukey’s HSD test (*p* < 0.05) was used for statistical analysis.

## 3. Results and Discussion

### 3.1. Microstructure

#### 3.1.1. FTIR Analysis

**Figure 2:**
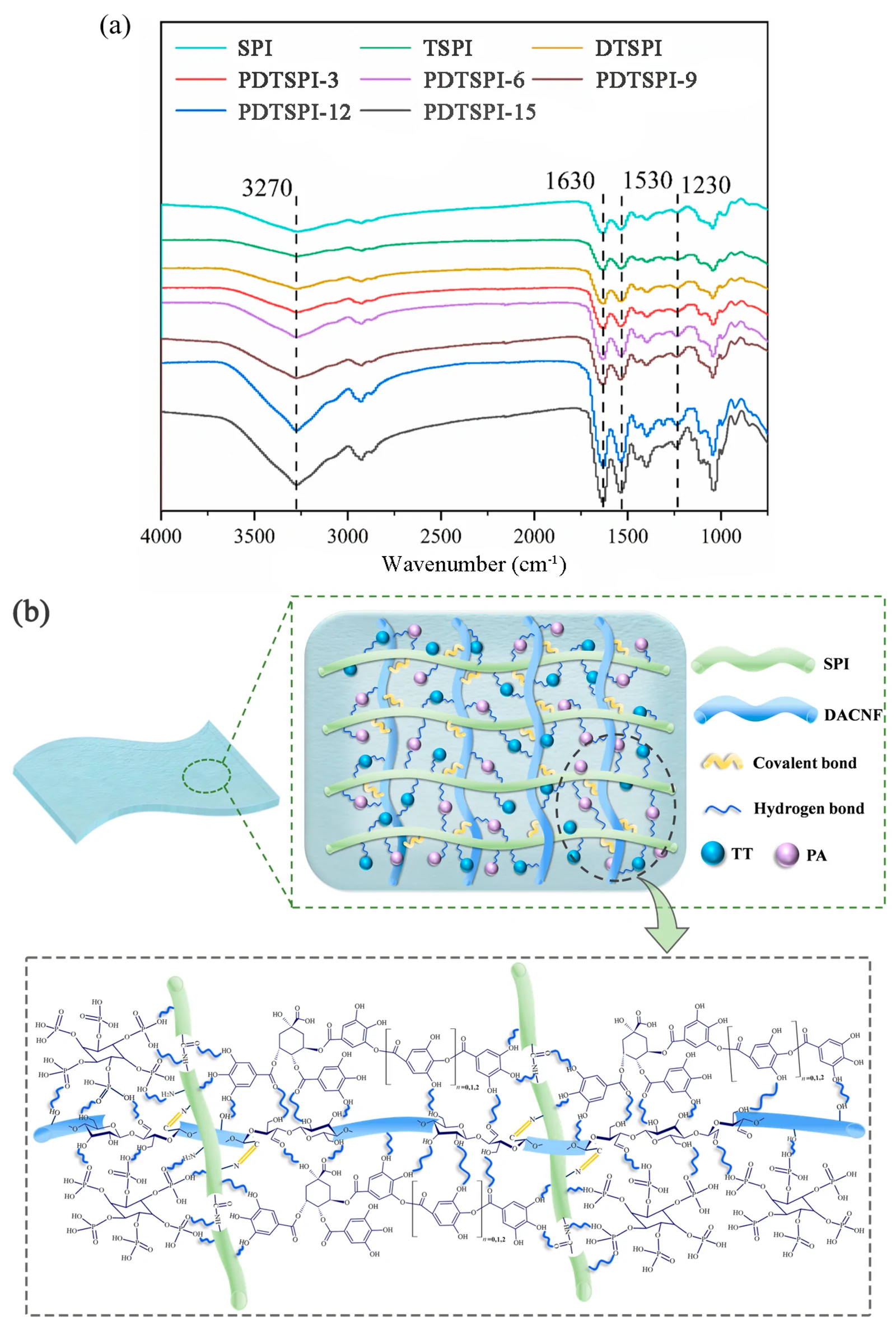
(**a**) FTIR spectroscopy of the films; (**b**) proposed mechanisms of PDTSPI films.

As presented in Figure 2a, the absorption peak at 3270 cm ^−1^ was attributed to the stretching vibration of O-H and N-H [28], and the peaks at 1630 cm^−1^, 1530 cm^−1^ and 1230 cm^−1^ were related to amide I (stretching vibration of C=O), amide II (bending vibration of N-H) and amide III (C-N stretching and N-H bending), respectively [29]. In our previous study, TT formed stable complexes with SPI and DACNF through hydrogen bonding interactions. Meanwhile, a Schiff base reaction occurred between SPI and DACNF to generate C=N bonds, which further enhanced the structural stability of the composite film [12]. Compared with the DTSPI spectrum, all PDTSPI samples displayed a broader absorption peak at 3270 cm^−1^; a slight red shift of the amide I band and a minor blue shift of the amide II band can also be observed. These changes might be related to the hydrogen bonds forming between phosphate groups in PA and amino groups in SPI [30]. However, the spectra of PDTSPI films were similar to those of DTSPI films. It could be inferred that PA caused the improved overall performance mainly due to hydrogen-bonding interactions instead of initiating new reactions. As a result, the synergistic effect of covalent and hydrogen bonds among SPI, TT, DACNF and PA formed a more stable and intricate crosslinking structure to further optimize the mechanical and functional properties. The proposed mechanism for the PDTSPI network structure is illustrated in Figure 2b.

#### 3.1.2. SEM Analysis

**Figure 3:**
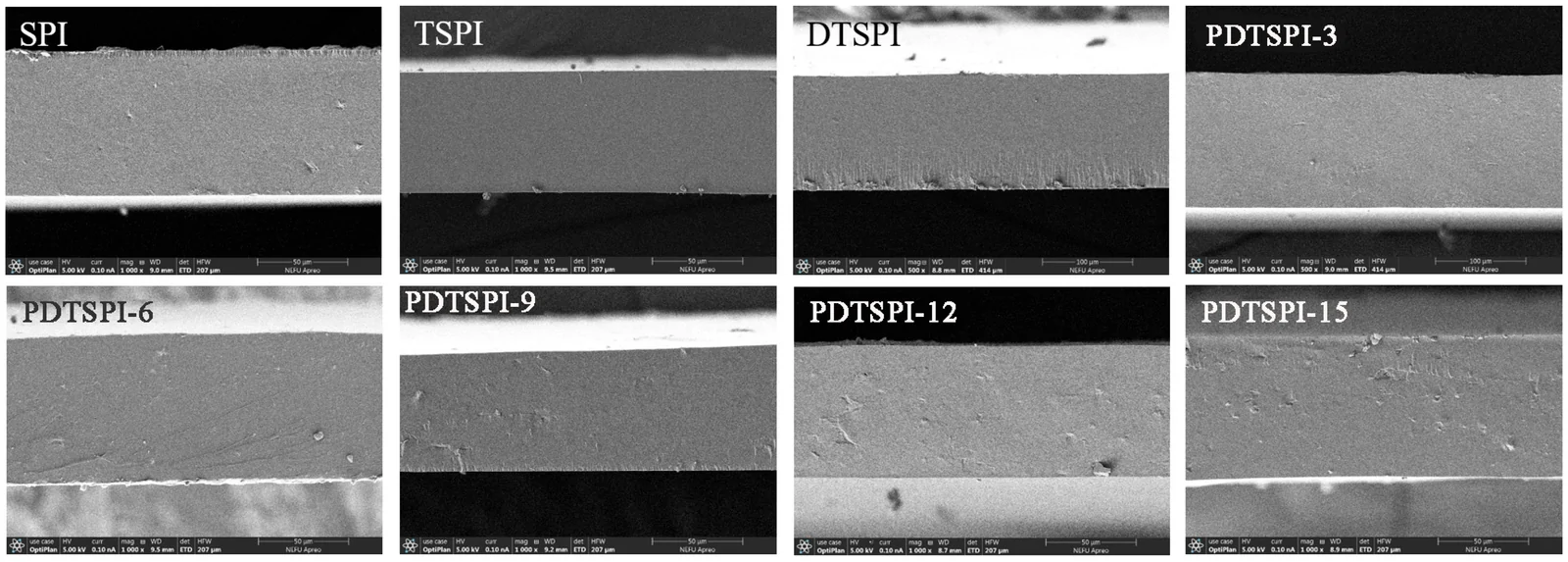
Cross-sectional SEM morphologies of various SPI films.

As shown in Figure 3, the cross-sectional morphology of the SPI film was smooth and continuous, indicating the excellent film-forming property of SPI. After the incorporation of TT and DACNF, the cross-section became denser and more homogeneous. The addition of PA did not change the micro-morphology evidently, demonstrating its good compatibility with SPI and other components. At 3% PA content, the cross-section of PDTSPI-3 film exhibited the best compactness, which was ascribed to the synergistic effect between hydrogen and covalent bonds achieving a balance [31]. Nevertheless, at higher PA concentrations (12% and 15%), micropores appeared in the cross-section, likely due to excessive chemical crosslinking forming a more rigid network structure and causing an accumulation of internal stress [32], which disrupted the stability of the film structure.

### 3.2. Physicochemical Properties of the Films

#### 3.2.1. Optical Properties

**Figure 4:**
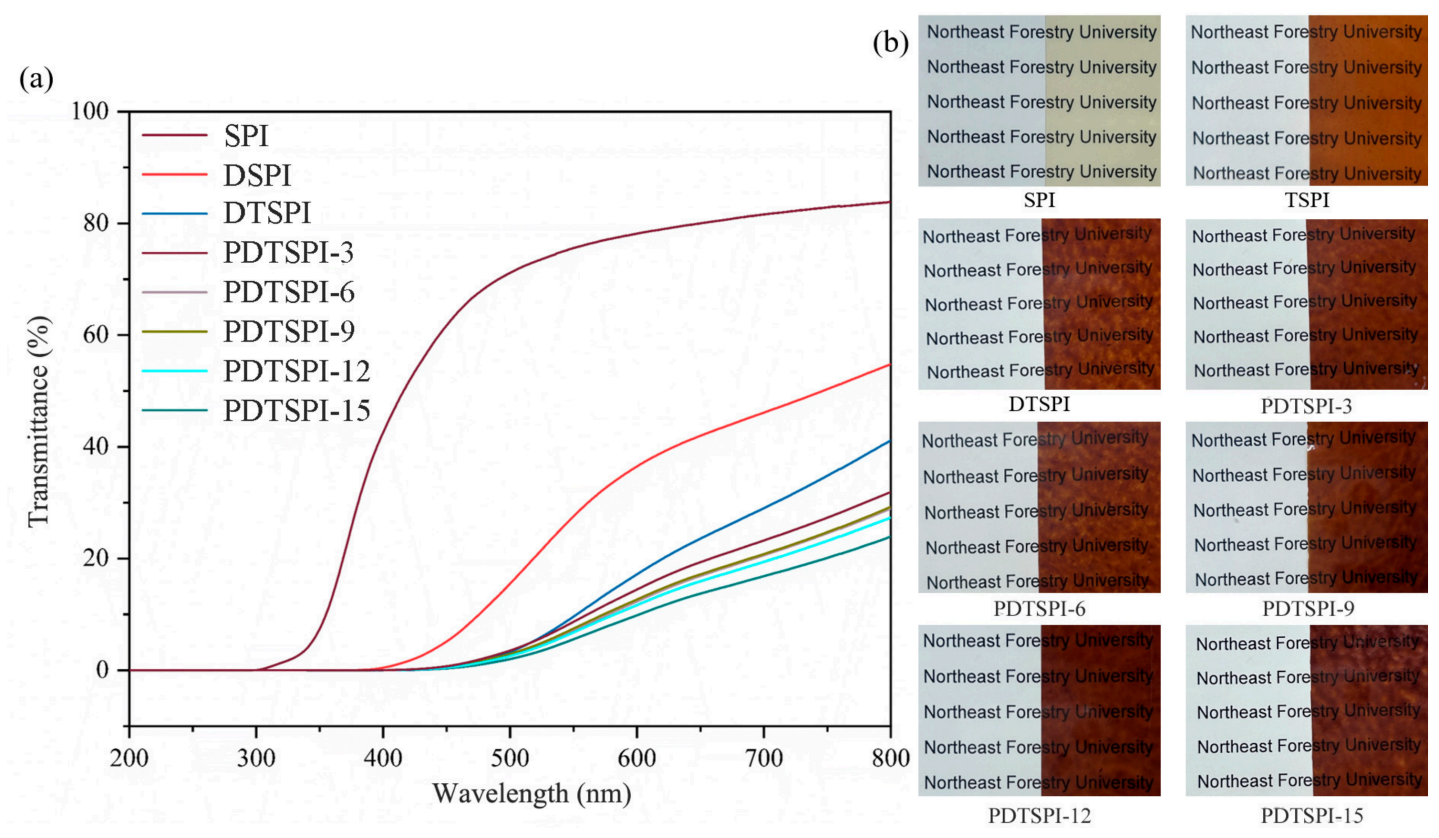
(**a**) UV–visible spectrums and (**b**) photographs of the film with different PA addition.

UV–Vis spectra and different colors of the films are presented in Figure 4a and Figure 4b, respectively. The SPI film showed the highest transmittance and only blocked UVA (320–400 nm) partly, especially in visible light regions, with a transmission value of nearly 80%. All composite films exhibited a significantly improved UV-blocking property in ultraviolet regions (200–400 nm) due to the polyphenol-conjugated structures of TT and complex crosslinking interactions among the components [33], and this phenomenon is consistent with a previous study focusing on red dragon fruit–soy protein isolate biofilm [34]. After the incorporation of PA, the light transmittance of SPI films was nearly 0% for wavelengths below 460 nm, which the visible light transmittance decreased to less than 40%. The optical barrier performance was improved compared with that reported in a previous study [11]. Furthermore, visible light transmittance decreased and the color of the SPI films became darker, gradually with increasing PA content from 0% to 15%, which might be ascribed to PA forming hydrogen bonds with SPI and DACNF. The more compact structure of the composite films enhanced light-scattering capacity and hindered the penetration of visible light [15]. Therefore, PDTSPI films could effectively mitigate the adverse effects of photo-oxidation on food preservation.

#### 3.2.2. Mechanical Properties

**Figure 5:**
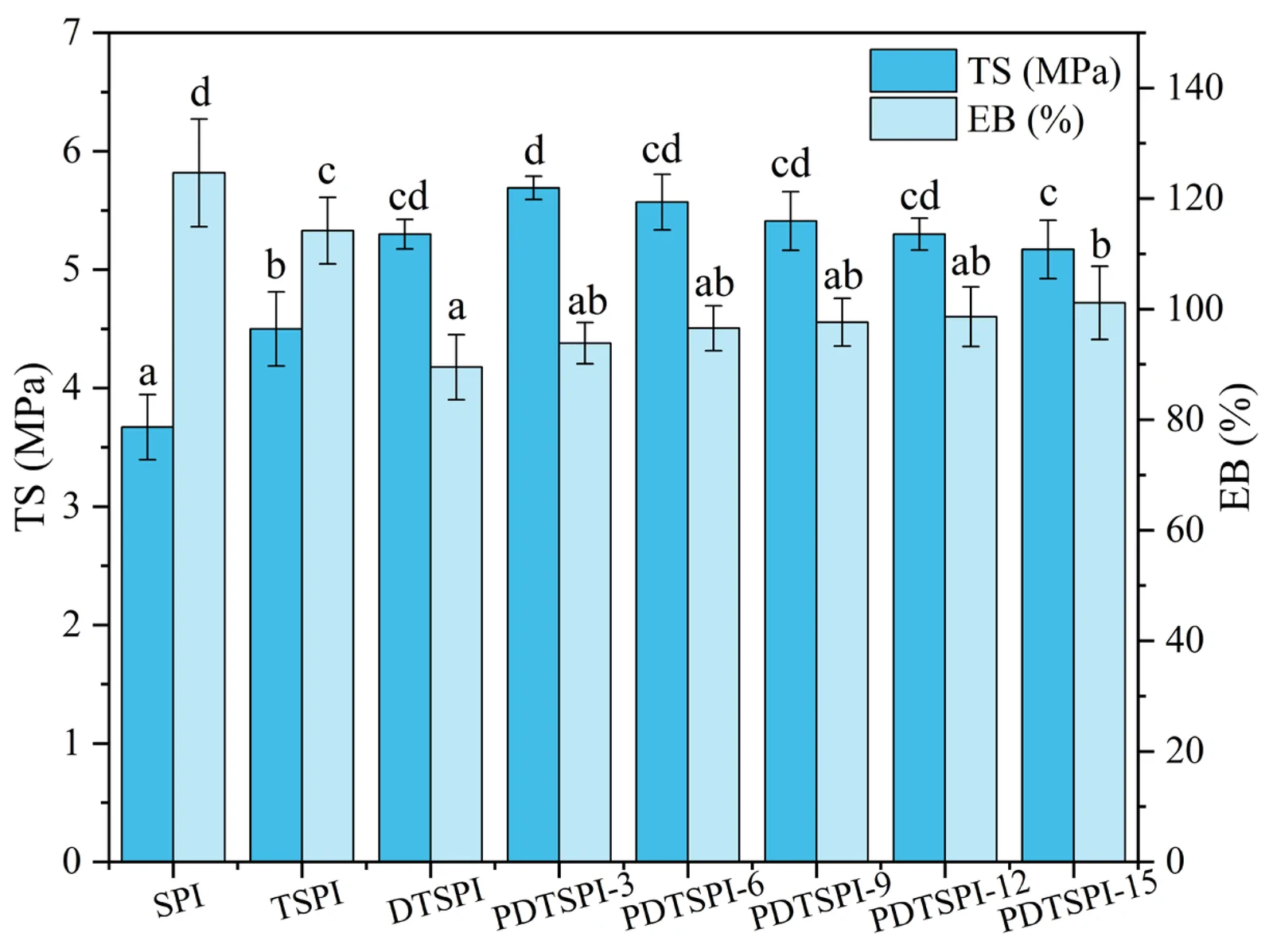
Mechanical performance of composite films. Error bars represent the standard deviation (SD) of parallel replicates; different lowercase letters above bars indicate significant differences (*p* < 0.05) according to Tukey’s HSD test.

The mechanical properties of different SPI films are illustrated in Figure 5. Hydrogen bonds between TT and SPI enhanced the intermolecular force; as a result, the tensile strength (TS) increased to 4.50 MPa, while the elongation at break (EB) decreased to 114.20%. The added DACNF generated covalent bonds with SPI to further limit the movements of the molecular chains [35], and the DTSPI film showed TS and EB of 5.30 MPa and 89.50%. As the PA content increased from 3% to 15%, the TS decreased from 5.69 MPa to 5.17 MPa, and the EB increased from 93.83% to 101.15%, which was ascribed to PA acting as a plasticizer [36]. Phosphate groups in PA formed hydrogen bonds and electrostatic interactions with amino groups in SPI molecules, which disrupted the original strong force between SPI molecules (such as intermolecular hydrogen bonds) and improved the chain mobility [37]. Compared with TSPI and DTSPI, PDTSPI-15 exhibited a comprehensive mechanical performance with a balance between TS and EB without inorganic nanoparticle fillers [38], manifesting that PDTSPI-15 is suitable for applying in food transportation and packaging preservation.

#### 3.2.3. Barrier Performance

Excellent oxygen and water vapor barrier performance effectively inhibited lipid oxidation as well as the growth and reproduction of bacteria and fungi. The water vapor permeability (WVP) and oxygen permeability (OP) of different films are listed in Table 1. WVP and OP of the pure SPI film were 1.82 g·m^−1^·s^−1^·Pa^−1^ × 10^−10^ and 2.27 cm^3^·mm·m^−2^·day^−1^·atm^−1^ × 10^−9^, respectively, demonstrating unsatisfactory barrier performance. This is because hydrogen-bonding interactions between polar groups in SPI and water molecules endow the SPI film with hydrophilic property [39]. Furthermore, it was difficult to form a tight structure, since weak interactions between protein molecules allowed oxygen to penetrate easily [40]. With the incorporation of TT and DACNF, an ordered crosslinking structure formed among molecular chains, boosting the barrier capacity of the films. The WVP and OP of the DTSPI film reduced to 1.65 g·m^−1^·s^−1^·Pa^−1^ × 10^−10^ and 1.07 cm^3^·mm·m^−2^·day^−1^·atm^−1^ × 10^−9^, respectively. At a low PA content (PDTSPI-3), PA crosslinked with amino acid residues in SPI to construct a tighter and denser structure, which could restrict the diffusion of water vapor and oxygen, further improving the barrier performance of the composite films. Nevertheless, as the PA content increased, the WVP and OP values showed a slightly rebound, higher than the PVA/SPI/Xanthoceras sorbifolia husk extract composite film [41]. This trend might be explained by the introduction of excess hydroxyl groups adsorbing environmental water molecules, which loosened the intermolecular packing arrangement. Over-crosslinking also diminished the structural compactness and improved oxygen and water vapor permeation rates.

#### 3.2.4. Antioxidant Activity

**Figure 6:**
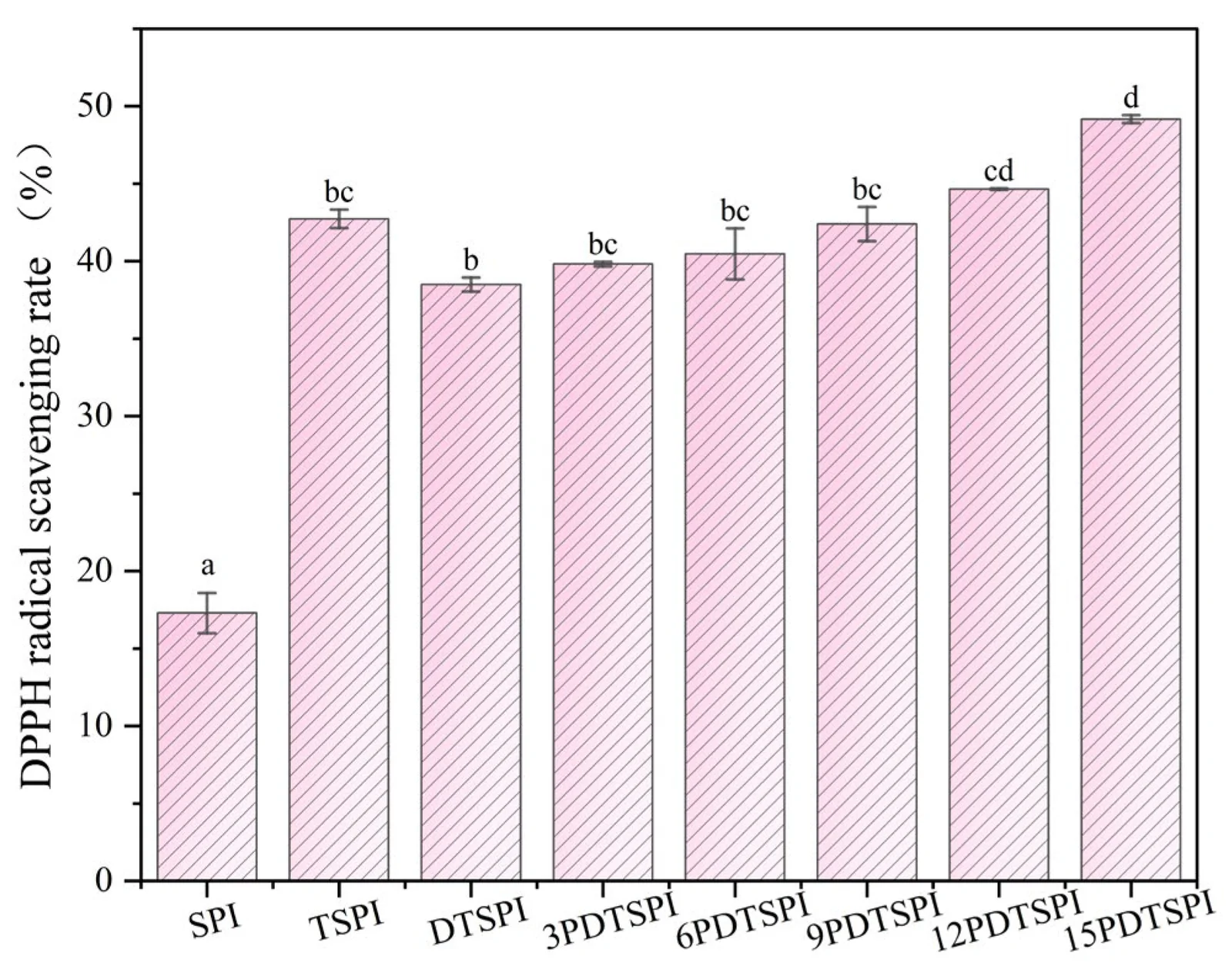
Effect of different PA additions on DPPH radical scavenging rate of composite films. Error bars represent the SD of parallel replicates; different lowercase letters above bars indicate significant differences (*p* < 0.05) according to Tukey’s HSD test.

The DPPH radical scavenging rate serves as a vital index to assess the antioxidant property of films. As illustrated in Figure 6, the pure SPI film exhibited the lowest DPPH SA, only 17.3%, due to the lack of hydrogen atom donors. The incorporation of TT provided abundant hydrogen donors and free electrons to inhibit DPPH radical activity [42], increasing the radical scavenging rate of TSPI films to 42.8%. DACNF itself could not act as a hydrogen donor, and physical crosslinking between DACNF and TT restricted the contact between tannin molecules and free radicals. Therefore, the antioxidant activity of DTSPI composite films was slightly reduced. However, with the amount of PA increased, improved DPPH scavenging activity could be observed. At 15% PA content, the DPPH radical scavenging rate peaked at 49.2%, which is higher than the value reported for the Xanthoceras sorbifolia husk extract-modified SPI film [9]. There are multiple electron-donating phosphate groups in PA, and when PA reacted with DPPH radicals, phosphate groups could provide electrons to eliminate free radicals [43]. Thus, PDTSPI exhibited an enhanced antioxidant property, endowing it with broad application prospects in packaging foods susceptible to oxidation and other related fields.

#### 3.2.5. Antibacterial Activity

**Figure 7:**
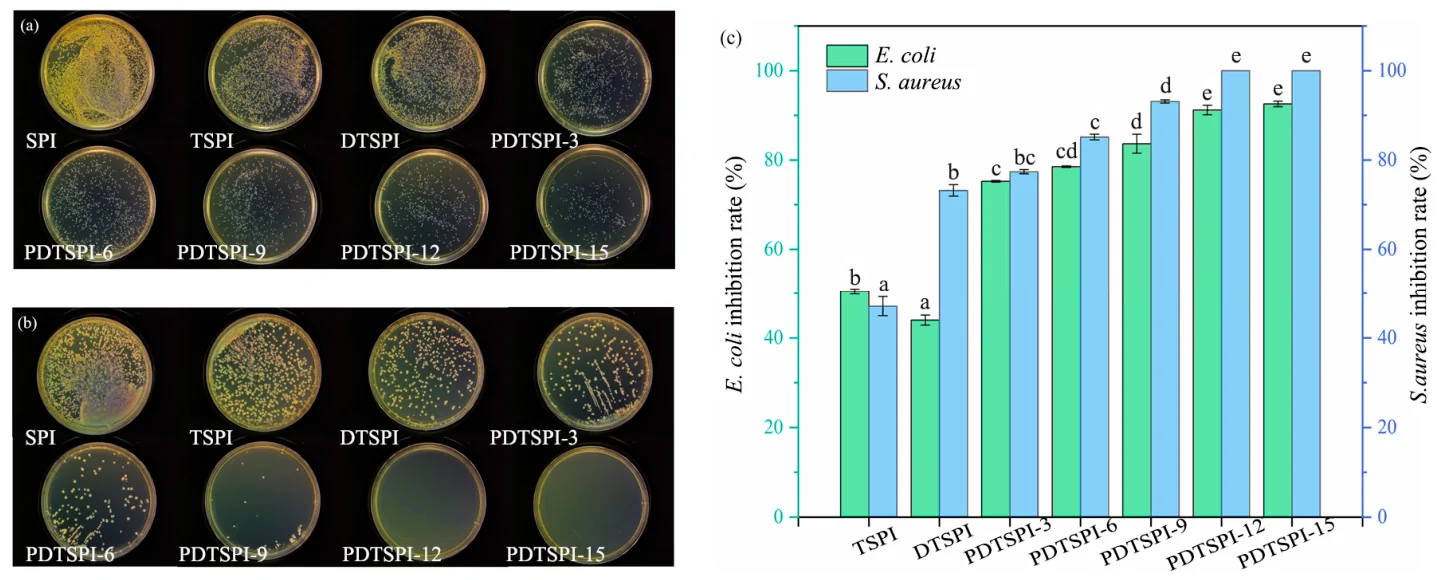
Antibacterial activity of the films against (**a**) *E. coli* and (**b**) *S. aureus*; (**c**) inhibition rate of films. Error bars represent the SD of parallel replicates; different lowercase letters above bars indicate significant differences (*p* < 0.05) according to Tukey’s HSD test.

*E. coli* and *S. aureus* were chosen as the standard strains for evaluating the antibacterial activity of films. As presented in Figure 7a,b, pure SPI films exhibited negligible antibacterial property. After incorporating TT and DACNF, the phenolic hydroxyl groups in TT and Schiff base derivatives improved the antibacterial activity of TSPI and DTSPI films [44]. Nevertheless, these composite films remained unsuitable for food packaging application. However, with the PA content increasing, antimicrobial effects on *E. coli* and *S. aureus* became more noteworthy. As shown in Figure 7c, the PDTSPI-12 and PDTSPI-15 films achieved a 100% inhibition rate against *S. aureus*, which could be related to abundant phosphate groups in PA. These phosphate groups interacted with protein and lipid molecules on cell membranes, resulting in the disruption of membrane integrity and the leakage of intracellular contents, thereby inhibiting bacterial metabolism and reproduction [45]. Compared with the SPI/thyme/mangosteen peel extract system with inhibition rates against *E. coli* and *S. aureus* of 95.79% and 92.63% [46], our PDTSPI-15 film exhibits better antibacterial effects. The findings confirmed that, as a natural and non-toxic bioactive substance, PA has potential application in antibacterial film material.

### 3.3. Temperature Affecting the Heat-Sealing Strength

**Figure 8:**
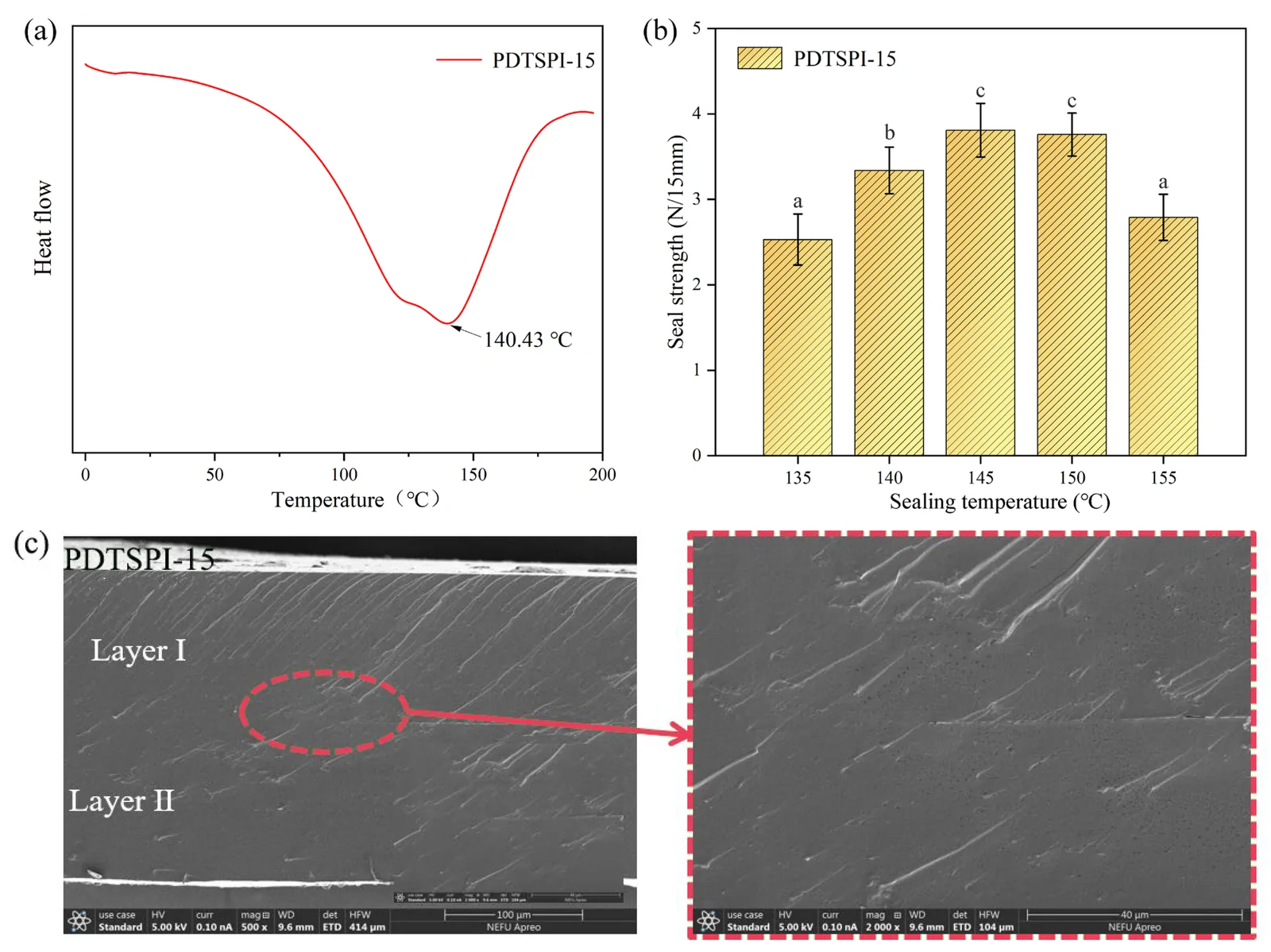
(**a**) DSC curve of PDTSPI-15; (**b**) heat-sealing strength of PDTSPI-15 at different temperatures; (**c**) SEM image of heat-sealed cross-section of PDTSPI-15. Error bars represent the SD of parallel replicates; different lowercase letters above bars indicate significant differences (*p* < 0.05) according to Tukey’s HSD test.

A DSC curve was used to define the range of heat-sealing temperatures. As illustrated in Figure 8a, PDTSPI-15 displayed an endothermic peak at approximately 140.43 °C, demonstrating the film in a melting state at this temperature range.

Referring to the phase transition temperature of PDTSPI-15 at 140.43 °C, the heat-sealing temperature range was set from 135 °C to 155 °C. As shown in Figure 8b, the maximum heat-sealing strength of the film reached 3.81 N/15 mm at 145 °C. Excessively high temperatures influenced the bond between two layers of the film and bubbles were formed in the heated parts, adversely making an impact on the heat-sealing effect. Temperatures lower than the optimal value would restrict the movement of molecular chains in the film matrix, inducing weak interfacial bonding and reduced heat-sealing strength. These results revealed that PDTSPI-15 exhibited optimum heat-sealing performance at 145 °C, which can serve as a reference temperature in food packaging applications.

Figure 8c presents cross-sectional SEM micrographs of the PDTSPI-15 film after heat sealing, and the tight interfacial adhesion without evident pores or voids could be observed. It might be attributed to the breakage and reformation of hydrogen bonds at contact surfaces [21], which facilitated compact interlayer bonding and endowed the PDTSPI-15 with superior heat-sealing performance.

### 3.4. Application in Beef Tallow and Chicken Preservation

#### 3.4.1. POV Analysis

Table 2 summarizes the effects on the peroxide value (POV) of beef tallow during storage. After 90 days of storage, the POV values of unpackaged beef tallow, packaged with conventional PE films, and pure SPI film beef tallow reached 0.694 g/100 g, 0.035 g/100 g and 0.015 g/100 g, respectively. This indicated that PE and SPI films remained with a limited antioxidant capacity. In comparison, the POV values of beef tallow packaged with DTSPI and PDTSPI-15 films were maintained at the lowest level of approximately 0.007–0.008 g/100 g, effectively inhibiting oxidative rancidity of fats and oils. This outstanding performance might be related to the incorporation of DACNF, which promoted the structural compactness and enhanced gas barrier properties of the films, thereby slowing down the lipid oxidation rate [47]. Moreover, TT possesses free radical scavenging activity, which is capable of inhibiting the lipid oxidative chain reaction and reducing the generation of peroxides. And the introduction of PA further improved the antioxidant property of PDTSPI-15 films, collectively, benefiting from the enhancement effect of DACNF as well as the synergistic antioxidant activity of TT and PA, making PDTSPI-15 film a highly promising material for oil-rich food packaging.

#### 3.4.2. TVC Analysis

**Figure 9:**
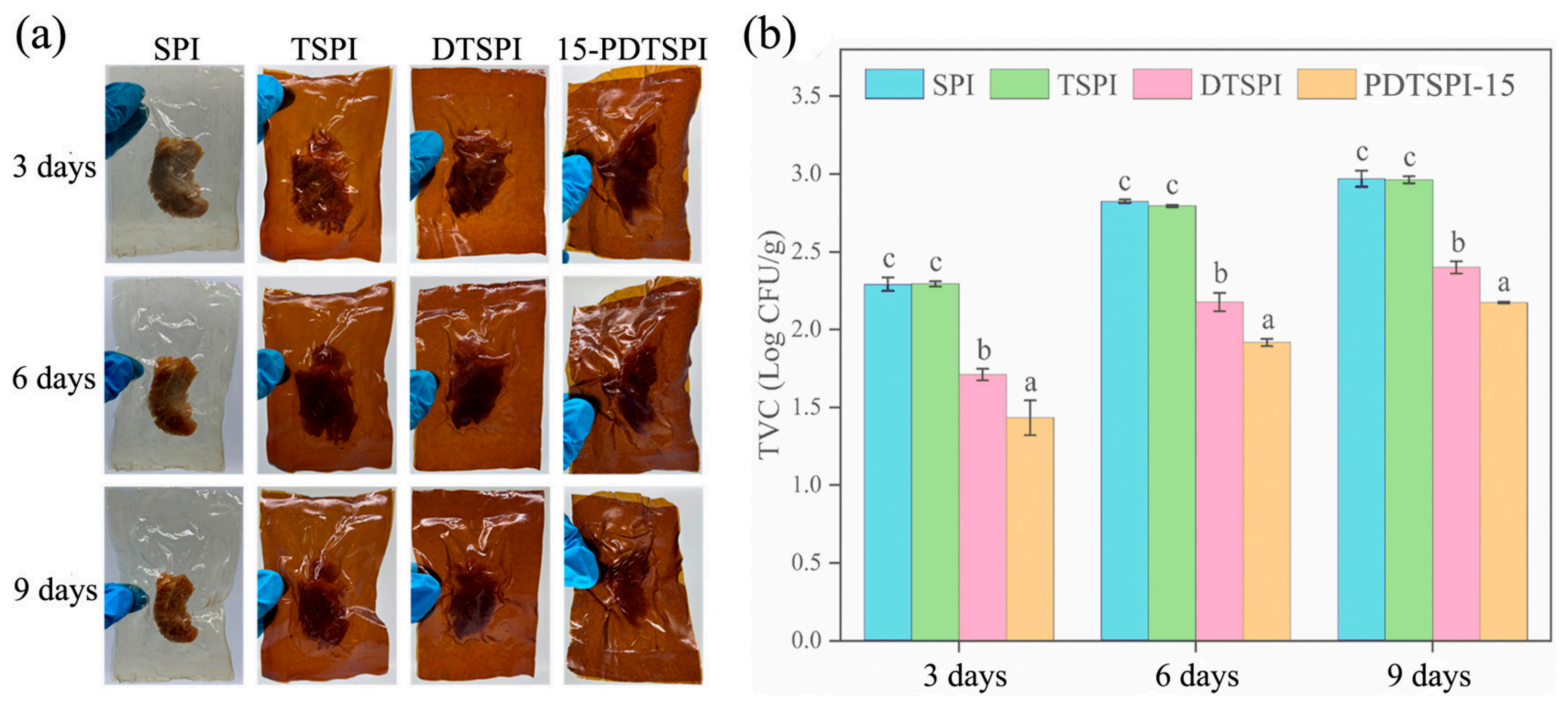
(**a**) Actual photographs and (**b**) changes in TCV of chicken under different packages. Error bars represent the SD of parallel replicates; different lowercase letters above bars indicate significant differences (*p* < 0.05) according to Tukey’s HSD test.

Figure 9 displays actual photographs and the changes in total viable count (TVC) of chicken under different packaging conditions. The experimental results showed that rapid bacterial proliferation occurred in chicken samples packaged with SPI and TSPI films, accompanied by a prominent increase in TVC values with prolonged storage duration. In contrast, after 9 days, the TVC values of samples packaged with DTSPI and PDTSPI-15 films were only 2.4 Log CFU/g and 2.17 Log CFU/g, respectively, which were lower than that of the pure SPI group (2.97 Log CFU/g). It could be explained by the fact that Schiff base derivatives and PA can disrupt and penetrate the cell wall and membrane of bacteria, hindering bacterial metabolism and proliferation [48], consequently endowing PDTSPI-15 films with excellent antibacterial efficiency.

## 4. Conclusions

In our study, PA was incorporated into the DACNF/TT/SPI system to prepare a composite film with improved antioxidant and antibacterial properties. FTIR revealed that PA formed hydrogen bonds with other materials to construct a more stable crosslinking structure, and SEM showed that the low PA content could optimize the compactness of cross-section. Moreover, this work represented reduced light transmittance in the ultraviolet and visible regions. Compared with DTSPI, the PDTSPI-15 exhibited the balanced mechanical performance, and the EB increased by 10.2%, but barrier properties against oxygen and water vapor should be further improved. In addition, the antioxidant and antimicrobial properties were impressive: the PDTSPI-15 film showed that the DPPH radical scavenging rate increased to 49.2%, and the inhibition rates for *E. coli* and *S. aureus* were 100% and 97.5%, respectively. In practical applications, the POV value of beef tallow packaged with PDTSPI-15 remained 0.007 g/100 g after 90 days. Meanwhile, the TVC value of packaged chicken was only 2.17 Log CFU/g after 9 days, demonstrating that PDTSPI-15 films can effectively delay oxidation and deterioration. In conclusion, this work indicates the great potential of PDTSPI films as eco-friendly materials in the food packaging field.

## Figures and Tables

**Table 1:** Effect of different PA additions on barrier properties of composite films. Different lowercase letters within the same column indicate significant differences (*p* < 0.05) according to Tukey’s HSD test.

Sample	WVP (g·m^−1^·s^−1^·Pa^−1^ × 10^−10^)	OP (cm^3^·mm·m^−2^·Day^−1^·Atm^−1^ × 10^−9^)
SPT	1.82 ± 0.53 c	2.27 ± 0.02 f
TSPI	1.80 ± 0.1 bc	1.78 ± 0.02 e
DTSPI	1.65 ± 0.4 a	1.07 ± 0.04 ab
PDTSPI-3	1.62 ± 0.67 a	0.98 ± 0.03 a
PDTSPI-6	1.66 ± 0.12 a	1.13 ± 0.04 b
PDTSPI-9	1.69 ± 0.07 ab	1.29 ± 0.06 c
PDTSPI-12	1.70 ± 0.35 abc	1.38 ± 0.02 cd
PDTSPI-15	1.73 ± 0.13 abc	1.47 ± 0.03 d

**Table 2:** POV value of beef tallow in different SPI films during storage. Different lowercase letters within the same column indicate significant differences (*p* < 0.05) according to Tukey’s HSD test.

Sample	POV (g/100 g)			
	Initial	30 Days	60 Days	90 Days
Unpackaged	0.004 ± 0 a	0.05 ± 0.001 d	0.082 ± 0.001 d	0.694 ± 0.01 c
PE	0.004 ± 0 a	0.012 ± 0 c	0.022 ± 0 c	0.035 ± 0 b
SPI	0.004 ± 0 a	0.008 ± 0 b	0.012 ± 0.001 b	0.015 ± 0 ab
DTSPI	0.004 ± 0 a	0.005 ± 0 a	0.007 ± 0 a	0.008 ± 0 a
PDTSPI-15	0.004 ± 0 a	0.005 ± 0 a	0.007 ± 0 a	0.007 ± 0 a

## Data Availability

The raw data supporting the conclusions of this article will be made available by the authors on request. Further inquiries can be directed to the corresponding author.

## References

[1] Kan M., Miller S.A. (2022). Environmental Impacts of Plastic Packaging of Food Products. Resour. Conserv. Recycl..

[2] Bora D., Hazarika K.K., Duarah R., Baruah S., Hazarika S. (2026). Advancement of Biodegradable Packaging for Sustainable Food Systems and Nutritional Security: Innovations, Challenges, and Global Perspectives. Renew. Sustain. Energy Rev..

[3] Qi Z., Wang B., Sun C., Yang M., Chen X., Zheng D., Yao W., Chen Y., Cheng R., Zhang Y. (2022). Comparison of Properties of Poly(Lactic Acid) Composites Prepared from Different Components of Corn Straw Fiber. Int. J. Mol. Sci..

[4] Galus S., Arik Kibar E.A., Gniewosz M., Kraśniewska K. (2020). Novel Materials in the Preparation of Edible Films and Coatings—A Review. Coatings.

[5] Mu Y., Lv S., Liu J., Tong J., Liu L., Wang J., He T., Wei D. (2025). Recent Advances in Research on Biomass-Based Food Packaging Film Materials. Compr. Rev. Food Sci. Food Saf..

[6] Yang X., Li Q., You Y., Li Y., Nian Y., Hu B. (2026). The Bioplastic Films Composed by Soybean Protein Isolate and Sodium Alginate for the Passively Modified Atmosphere Packaging and Preservation of Fruits and Vegetables. Colloids Surf. A.

[7] Wang K., Li X., Peng H., Dong Y., Li Y., Liu X., Li J. (2022). Tough and Strong Soy Protein Film by Integrating CNFs and MXene with Photothermal Conversion and UV-Blocking Performance. Cellulose.

[8] Cheng J., Gao R., Zhu Y., Lin Q. (2024). Applications of Biodegradable Materials in Food Packaging: A Review. Alex. Eng. J..

[9] Han Y., Yan W., Hou Y., Wang D., Yu M. (2023). Xanthoceras sorbifolia Husk Extract Incorporation for the Improvement in Physical and Antioxidant Properties of Soy Protein Isolate Films. Foods.

[10] Wu X., Wang M., Zheng H. (2026). Effect of Natural Polyphenols on the Structural and Properties of Soy Protein Isolate/κ-Carrageenan Composite Films: A Comparative Study. Food Hydrocoll..

[11] Ren Z., Ning Y., Xu J., Cheng X., Wang L. (2024). Eco-Friendly Fabricating Tara Pod Extract-Soy Protein Isolate Film with Antioxidant and Heat-Sealing Properties for Packaging Beef Tallow. Food Hydrocoll..

[12] Ren Z., Ning Y., Jiang A., Xu J., Cheng X., Wang L. (2025). Fabricating an Antioxidant and Bacteriostatic Soy Protein Isolate Film Double-Crosslinked via Dialdehyde Cellulose Nanofibers and Tara Tannins for Beef Tallow and Cooked Pork Preservation. Int. J. Biol. Macromol..

[13] Xu X., Deng S., Essawy H., Lee S.H., Lum W.C., Zhou X., Du G., Zhang J. (2024). Chitosan-Casein Blended with Condensed Tannin and Carnauba Wax for the Fabrication of Antibacterial and Antioxidant Food Packing Films. Int. J. Biol. Macromol..

[14] Gyawali R., Ibrahim S.A. (2014). Natural Products as Antimicrobial Agents. Food Control.

[15] Ning Y., Liu R., Chi W., An X., Zhu Q., Xu S., Wang L. (2024). A Chitosan Derivative/Phytic Acid Polyelectrolyte Complex Endowing Polyvinyl Alcohol Film with High Barrier, Flame-Retardant, and Antibacterial Effects. Int. J. Biol. Macromol..

[16] Canan C., Kalschne D.L., Ongaratto G.C., Leite O.D., Cursino A.C.T., Flores E.L.M., Ida E.I. (2021). Antioxidant Effect of Rice Bran Purified Phytic Acid on Mechanically Deboned Chicken Meat. J. Food Process. Preserv..

[17] Yin Q., Liu J., Zhong Z., Zhang Y., Zhang F., Wang M. (2023). Synthesis of Phytic Acid-Modified Chitosan and the Research of the Corrosion Inhibition and Antibacterial Properties. Int. J. Biol. Macromol..

[18] Ning Y., Liu R., Chi W., Liu W., Zhu Q., Xu S., Wang L. (2023). Simultaneously Flame-Retardant and Antibacterial Films from κ-Carrageenan/Carboxylated Cellulose Nanofibril/Phytic Acid for the Preservation of Cooked Pork. Food Hydrocoll..

[19] López O.V., Lecot C.J., Zaritzky N.E., García M.A. (2011). Biodegradable Packages Development from Starch Based Heat Sealable Films. J. Food Eng..

[20] Luo Y., Wang Y., Xia C., Ahmad A., Yang R., Li X., Shi S.Q., Li J., Guo M., Nadda A.K. (2022). Eco-Friendly Soy Protein Isolate-Based Films Strengthened by Water-Soluble Glycerin Epoxy Resin. Prog. Org. Coat..

[21] Lu J., Li T., Ma L., Li S., Jiang W., Qin W., Li S., Li Q., Zhang Z., Wu H. (2021). Optimization of Heat-Sealing Properties for Antimicrobial Soybean Protein Isolate Film Incorporating Diatomite/Thymol Complex and Its Application on Blueberry Packaging. Food Packag. Shelf Life.

[22] Xu Y., Xu Y., Deng W., Chen H., Xiong J. (2023). Extracting Dialdehyde Cellulose Nanocrystals Using Choline Chloride/Urea-Based Deep Eutectic Solvents: A Comparative Study in NaIO_4_ Pre-Oxidation and Synchronous Oxidation. Int. J. Biol. Macromol..

[23] (2006). Plastics—Determination of Tensile Properties—Part 3: Test Conditions for Films and Sheets.

[24] (2003). Standard Practice for Using ASTM E96 Test Methods to Determine Water Vapor Transmission of Exterior Insulation and Finish Systems (EIFS).

[25] (2021). Packaging Material—Test Method for Oxygen Gas Permeability Characteristics of Plastic Film and Sheeting—Coulometric Sensor.

[26] (2023). National Food Safety Standard—Determination of Peroxide Value in Foods.

[27] (2022). National Food Safety Standard—Food Microbiological Examination—Aerobic Plate Count.

[28] Yadav M., Behera K., Chang Y.-H., Chiu F.-C. (2020). Cellulose Nanocrystal Reinforced Chitosan Based UV Barrier Composite Films for Sustainable Packaging. Polymers.

[29] Tummino M.L., Chrimatopoulos C., Bertolla M., Tonetti C., Sakkas V. (2023). Configuration of a Simple Method for Different Polyamides 6.9 Recognition by ATR-FTIR Analysis Coupled with Chemometrics. Polymers.

[30] Pi X., Liu J., Sun Y., Ban Q., Liang S., Cheng J., Guo M. (2023). Effect of Proanthocyanidins on Protein Composition, Conformational Structure, IgE Binding Capacities and Functional Properties in Soybean Protein. Int. J. Biol. Macromol..

[31] Cheng L., Lian Z., Liu X., Dai S., Li L., Wang M., Li K., Ren K., Tong X., Wang H. (2024). Effect of Phlorotannins Modification on the Physicochemical, Structural and Functional Properties of Soybean Protein Isolate and Controlled Hydrolysates: Covalent and Non-Covalent Interactions. Food Hydrocoll..

[32] Wang X., Huang Z., Niu Z., Chen F., Liu C. (2022). Glutaraldehyde Crosslinked High Content of Amylose/Polyvinyl Alcohol Blend Films with Potent Tensile Strength and Young’s Modulus. Polymers.

[33] Zhang Y., Jiang W. (2023). Effective Strategies to Enhance Ultraviolet Barrier Ability in Biodegradable Polymer-Based Films/Coatings for Fruit and Vegetable Packaging. Trends Food Sci. Technol..

[34] Prajapati R.A., Jadeja G.C. (2024). Red dragon fruit-soy protein isolate biofilm: UV-blocking, antioxidant & improved mechanical properties for sustainable food packaging. J. Food Sci. Technol..

[35] Xu Y., Fu L., Cao F., Sun C., Fang Z., Qiao Z. (2024). Preparation of Strong Soy Protein Composite Materials with Thermal Conductivity and Biodegradability by Constructing Nanophase Enhanced Organic-Inorganic Hybrid Structure. Ind. Crops Prod..

[36] Liu Y., Wang J., Yue H., Du Z., Cheng X., Wang H., Cheng F., Du X. (2024). Flame-Retardant Phytic Acid–Decorated Thermoplastic Starch/Halloysite Nanotube Composite Films with Enhanced Mechanical Strength and Excellent Barrier Properties. Carbohydr. Polym..

[37] Amat T., Assifaoui A., Buczkowski J., Silva J.V.C., Schmitt C., Saurel R. (2024). Interplay between Soluble and Insoluble Protein/Calcium/Phytic Acid Complexes in Dispersions of Faba Bean and Pea Protein Concentrates around Neutral pH. Food Hydrocoll..

[38] Havanur S.G., Pinto J.P., Priyadarshini, Baburao P., Sheeparamatti B.A., Chougale R.B., Hunashyal A.A., Masti S.P., Gonal N., Arakera S.B. (2026). Development of ortho silicic acid crosslinked sodium alginate/PVOH blend films reinforced with biosynthesised silver nanoparticles: Sustainable approach for the preservation of chicken meat. BioNanoScience.

[39] Pang H., Zhao S., Qin T., Zhang S., Li J. (2019). High-Performance Soy Protein Isolate-Based Film Synergistically Enhanced by Waterborne Epoxy and Mussel-Inspired Poly(dopamine)-Decorated Silk Fiber. Polymers.

[40] Sundaresan K.V., Ludescher R.D. (2008). Molecular Mobility and Oxygen Permeability in Amorphous β-Lactoglobulin Films. Food Hydrocoll..

[41] Yan W., Han Y., Hou Y., Wang D., Yu M. (2023). Effects of polyvinyl alcohol incorporation on the physical and antioxidant properties of soy protein isolate/*Xanthoceras sorbifolia* husk extract active films. Food Biosci..

[42] Li J., Zheng Y., Wang P., Zhang H. (2024). The Alginate Dialdehyde Crosslinking on Curcumin-Loaded Zein Nanofibers for Controllable Release. Food Res. Int..

[43] Chen Y., Sha R., Dai J., Wang Z., Mao Y., Fan F., Huang J., Mao J. (2025). Phytic Acid Extraction from Rice Bran with Acetic Acid: Optimization, Antioxidant Capacity and Mechanism Based on Cellular Metabolomics. LWT-Food Sci. Technol..

[44] Long T., Xu T., Li R., Xu Z., Li D., Mu C., Yuan L., Mu Y. (2024). Emulsion Template Fabricated Gelatin-Based Scaffold Functionalized by Dialdehyde Starch Complex with Antibacterial Antioxidant Properties for Accelerated Wound Healing. Int. J. Biol. Macromol..

[45] Zhou Q., Zhao Y., Dang H., Tang Y., Zhang B. (2019). Antibacterial Effects of Phytic Acid against Foodborne Pathogens and Investigation of Its Mode of Action. J. Food Prot..

[46] Lu Y., Huang Y., Zhang T., Zhou X., Li H., Qin Z. (2022). Fabrication of anti-UV absorbing and antibacterial soybean protein isolate composite film modified with thyme and mangosteen peel extracts. J. Appl. Polym. Sci..

[47] Vilarinho F., Stanzione M., Buonocore G.G., Barbosa-Pereira L., Sendón R., Vaz M.F., Sanches Silva A. (2021). Green Tea Extract and Nanocellulose Embedded into Polylactic Acid Film: Properties and Efficiency on Retarding the Lipid Oxidation of a Model Fatty Food. Food Packag. Shelf Life.

[48] Hamed A.A., Abdelhamid I.A., Saad G.R., Elkady N.A., Elsabee M.Z. (2020). Synthesis, Characterization and Antimicrobial Activity of a Novel Chitosan Schiff Bases Based on Heterocyclic Moieties. Int. J. Biol. Macromol..

